# Supplementary material for: Setup uncertainties and appropriate setup margins in the head-tilted supine position of whole-brain radiotherapy (WBRT)
Source: PLoS One. 2022 Aug 4;17(8):e0271077. doi: 10.1371/journal.pone.0271077 (PMC9352041; doi:10.1371/journal.pone.0271077)
Supplement: Attachment — Submitted filename: Headtilt_manuscript (1)_ZJreview.docx [file pone.0271077.s001.docx]

Setup uncertainties and appropriate setup margins in the head-tilted supine position of whole-brain radiotherapy (WBRT)

Short Title: Head-tilted supine position of whole-brain radiotherapy(WBRT) setup

Jae Won Park ^1,2^, Ji Woon Yea ^1,2^, Jaehyeon Park ^1,2^, Se An Oh ^1,2*^

^1^ Department of Radiation Oncology, Yeungnam University Medical Center, Daegu, Korea

^2^ Department of Radiation Oncology, Yeungnam University College of Medicine, Daegu, Korea

* Corresponding author:
Se An Oh
E-mail: [sean.oh5235@gmail.com](mailto:sean.oh5235@gmail.com) (SAO)

Abstract

This study evaluated the setup uncertainties and determined the appropriate planning target volume (PTV) margins for patients treated with and without a head-tilting baseplate in the conventional supine position in whole-brain radiotherapy (WBRT). Thirty patients who received WBRT at our institution between October 2020 and May 2021 in the conventional supine and head-tilted supine positions were investigated. The DUON head mask (60124, Orfit Industries, Wijnegem, Belgium) was used in the conventional supine position, and a thermoplastic U-Frame Mask (R420U, Klarity Medical & Equipment Co. Ltd., Lan Yu, China) was used in the head-tilted supine position. Radiotherapy was performed by correcting the translational (lateral, longitudinal, and vertical) and rotational (yaw) errors based on the differences in computed tomography (CT) and cone-beam CT. The PTV margins were calculated using Stroom et al.’s [2Σ+0.7σ] and van Herk et al.’s recipe [2.5Σ+0.7σ]. Appropriate PTV margins with van Herk et al.’s recipe in WBRT were < 2 mm, 1.5° in the supine position, and < 3 mm and 2° in the head-tilted supine position in the translational and rotational directions, respectively. Although the head tilt in the supine position requires more margin, it can be applied as a sufficiently stable and effective position in radiotherapy.

# Introduction

Radiotherapy for patients with brain tumours are based on various factors, such as the size, location, and cell type of the primary tumour. Whole-brain radiotherapy (WBRT) is a commonly used effective technique to relieve neurological symptoms, improve the quality of life, and prolong survival **[1]**,**[2]**,**[3]**,**[4]**,**[5]**.

In our previous study **[6]**, we have demonstrated that head-tilting techniques can be effectively used to reduce the radiation dose entering normal tissues, such as the hippocampus, lens, and parotid gland, using volumetric arc therapy (VMAT). Moreover, the radiation conformity and dose distribution in tumours simultaneously improved.

Recently, various studies using a head-tilting baseplate in radiotherapy have been published. Miura et al. **[7]** compared the potential of the use of tomotherapy in the dose distribution improvement of the planning target volume (PTV) and the exposure time reduction of normal organs, such as the hippocampus and lens with and without the head-tilting baseplate in hippocampal-sparing WBRT. Tilted hippocampal-sparing WBRT reportedly reduced the irradiation time by more than 10% during sparing of normal organs, such as the hippocampus and lens.

Shimizu et al. **[8]** used the four-field box technique in WBRT to examine the best head-tilt angle that could reduce the parotid gland dose while maintaining a safe level of lens dose. Since the parotid dose is inversely proportional to the lens dose, it was concluded that the orbitomeatal plane angle required to reduce the maximum lens dose to less than 10 Gy and minimise the parotid gland dose was 14°.

Lin et al. **[9]** investigated the various head flexion angles in hippocampal-sparing WBRT in VMAT. This study demonstrated that at least 15° should be implemented in clinical practice, and for better dose coverage and uniformity of whole-brain PTV and dose reduction in critical organs, a head angle of ≥25° is recommended.

Therefore, the application of the head tilt in WBRT for radiotherapy has several advantages. However, a study on the setup uncertainties in head-tilting techniques in WBRT and the clinical target volume (CTV) to PTV setup margins has not been reported. Although the head-tilt technique has several advantages in radiotherapy, if the setup uncertainties are excessively large, the PTV margin becomes large and the dose delivered to the normal organs, such as the hippocampus, parotid, and lens, increases; thus, the existing advantages of the head-tilt technique may disappear. Therefore, it is necessary to study the exact setup uncertainties and PTV margins for the head-tilt technique compared to that of the existing supine setup.

Hence, this study evaluated the setup uncertainties and determined the appropriate PTV margins for patients treated with and without a head-tilting baseplate and in the conventional supine position in WBRT.

# Materials and Methods

## Study overview

This retrospective data analysis of the 30 patients who underwent WBRT enrolled in this study was approved by the Institutional Review Board of the Yeungnam University Medical Center (YUMC 2021-07-033). Informed consent was specially waived under the approval of the institutional review board, given that patient anonymity was ensured.

## Patient selection

All the patients who received WBRT between October 2020 and May 2021 at our institution were included in the study; 30 consecutive patients who received radiotherapy in the conventional supine and head-tilted supine positions fixed with a thermoplastic mask were investigated. The patient radiotherapy techniques and treatment schedules included in this study are described in Table 1. Of the 30 WBRT patients, 15 were treated using the conventional supine position and 15 patients in the head-tilted supine position. Patients treated in the supine position had one patient who received 25 Gy in 10 fractions, and 14 patients who received 30 Gy in 10 fractions for a total fraction number of 150. In addition, among the patients treated in the head-tilted supine position, one patient received 25 Gy in 10 fractions, and 14 patients who received 30 Gy in 10 fractions for a total fraction number of 150.

Table 1. Characteristics of the patients and treatment included in this study.

| **Patient characteristics** | |  |
| --- | --- | --- |
|  | Supine position | Head-tilted supine position |
| **Number of patients** | N=15 | N=15 |
| **Median age (Range)** | 59(35-83) | 62(37-86) |
| **Sex (%)** |  |  |
| **Female** | 9(60) | 7(46.7) |
| **Male** | 6(40) | 8(53.3) |
| **Radiotherapy techniques (%)** |  |  |
| **3D** | 2(13) | 15(100) |
| **IMRT** | 13(87) | 0(0) |
| **Treatment schedule** | |  |
| **Number of fractions** | n=150 | n=150 |
| **Fraction schemes (%)** |  |  |
| **25 Gy in 10 fractions** | 1(7) | 1(7) |
| **30 Gy in 10 fractions** | 14(93) | 14(93) |

3D=three-dimensional radiotherapy; IMRT=intensity-modulated radiotherapy

## Immobilisation and CT simulation

A thermalplastic mask was used in all the patients to minimise the inter-fractional and intra-fractional variations of radiotherapy. In the traditional supine position, the DUON head mask (60124, Orfit Industries, Wijnegem, Belgium) was fixed at 2.4-mm mask thickness. For the head-tilted supine position, a tilting acrylic supine baseplate (MedTec, USA) was used to elevate the patient’s head to up to 40° according to our institution’s protocol, and a thermoplastic U-Frame Mask (R420U, Klarity Medical & Equipment (GZ) Co. Ltd., Lan Yu, China) with 2.4-mm mask thickness was used. All the computed tomography (CT) simulation images were obtained using a Brilliance Big Bore CT simulator (Philips Inc., Cleveland, OH) with a thickness of 2.5–5 mm.

## Treatment planning and delivery techniques

An anisotropic analytic algorithm (AAA Varian Eclipse TPS, version 15.6.05) was used for all the radiation treatment plans; the treatment plan for WBRT has already been mentioned in previous studies [6], [10]. The PTV was created with an extension of 5 mm in all directions from the CTV. Three-dimensional radiotherapy (3D) uses a beam arrangement in 0°, 0°, 180°, and 270° directions, and the intensity-modulated radiotherapy (IMRT) technique uses VMAT with two coplanar full-arc beams. Fig 1 shows the setup position of the patient in the supine and the head-tilted supine position with the thermal mask before radiotherapy in the radiation treatment room.

**Fig 1. Patient setup position for radiotherapy.** (A) supine position, (B) head-tilted supine position

## Image registration and setup protocol

The image registration using planning CT and cone-beam CT (CBCT) in the conventional supine position and the head-tilted supine position is shown in Figs 2 and 3, respectively. Daily setup verification images were obtained for all the 30 patients who received WBRT using CBCT (Varian Medical Systems, Palo Alto, CA, USA) before treatment. Fig 2(a) shows the planning CT images in transverse, frontal, and sagittal directions; Fig 2(b) was obtained with CBCT in pre-treatment. Fig 2(c) shows the registration images between the planning CT and CBCT images in the translational (lateral, longitudinal, and vertical) and rotational (yaw) directions. Image registration between the planning CT and CBCT image was performed using bony anatomy matching. Radiation treatment was performed after correcting the translational (lateral, longitudinal, and vertical) and rotational (yaw) directions based on the differences in the planning CT and CBCT. The couch automatically corrects the setup difference in the translational (lateral, longitudinal, and vertical) and rotational (yaw) directions, the setup corrections were recorded.

**Fig 2. Image registration using the planning computed tomography (CT) and cone beam CT (CBCT) in the conventional supine position.** (A) Planning CT, (B) CBCT, and (C) Registration image.

**Fig 3. Image registration using the planning computed tomography (CT) and cone beam CT (CBCT) in the head-tilted supine position.** (A) Planning CT, (B) CBCT, and (C) Registration image.

## Analysis of the setup uncertainties between the planning CT and CBCT

Van Herk introduced a method to analyse the random errors ($\boldsymbol{\sigma}$) and systematic error ($\boldsymbol{\Sigma}$) using setup correction values for setup verification [11]. In addition, random errors ($\boldsymbol{\sigma}$) and systematic error ($\boldsymbol{\Sigma}$) have already been mentioned in several papers [12], [13]. During the image registration process, the differences in four directions, translational (lateral, longitudinal, and vertical) and rotational (yaw) directions, were analysed between the planning CT and CBCT images.

A one-sample Kolmogorov–Smirnov test was performed to verify the normal distribution of all the recovered setup corrections. All the data were not normally distributed in any direction. A non-parametric Mann–Whitney U-test was performed to compare the two independent groups between the supine position and the head-tilted supine position. All the statistical tests were performed using the SPSS software package (SPSS Inc., Chicago, IL, USA), and a *p*-value < 0.05 was considered statistically significant.

We also tried to compare the 3D vector values between the supine and head-tilted supine positions. The 3D vector values can be calculated as follows:

$$3D vector=\sqrt{x^{2}+y^{2}+z^{2}}$$

where x, y, and z are the errors in the lateral, longitudinal, and vertical directions, respectively.

## Calculation of the PTV margin by CTV expansion

Several studies [11],[12],[13],[14],[15],[16],[17],[18],[19],[20] have already been conducted on the appropriate PTV margin recipe through expansion from CTV. In this study, the PTV margin was calculated using the methods of Stroom et al. [14] and Van Herk et al. [15].

Stroom et al. [14] assumed a 95% dose, on an average of 99% of the CTV tested in a practical setting. In addition, Van Herk et al. assumed that the minimum dose for the CTV is 95% for 90% of the patients using the analytical solution for perfect conformation.

$$Stroom et al.^{'}s recipe=2\Sigma+0.7\sigma$$

$$Van Herk etl al.^{'}s recipe=2.5\Sigma+0.7\sigma$$

where $\boldsymbol{\Sigma}$ is the standard deviation of the systematic errors, and $\boldsymbol{\sigma}$ is the standard deviation of random errors.

# Results

Figs 4 and 5 show the histograms and normalised curves of the setup errors in translation (lateral, longitudinal, and vertical) and rotation (yaw) in the supine and head-tilted supine positions, respectively.

**Fig 4. Histograms and normalised curves of the setup errors in the translation and rotation for the supine position.** Setup errors in the (A) lateral, (B) longitudinal, (C) vertical, and (D) yaw directions.

**Fig 5. Histograms and normalised curves of the setup errors in the translation and rotation for the head-tilted supine position.** Setup errors in the (A) lateral, (B) longitudinal, (C) vertical, and (D) yaw directions.

Table 2 shows the systematic errors ($\Sigma$) and random errors ($\sigma$) in the translational and rotational directions for the supine and head-tilted supine positions. In the supine position, the mean values of the systematic error in the lateral, longitudinal, vertical, and yaw directions were -0.80 mm, 0.79 mm, 0.37 mm, and 0.17°, respectively, and the standard deviation values of the systematic error were 0.47, 0.34, 0.54, and 0.56, respectively. The mean values of the random error were 0.27 mm, 0.54 mm, 0.39 mm, and 0.35°, respectively, and the standard deviation values of the random errors were 0.24, 0.31, 0.20, and 0.17, respectively. On the other hand, the mean values of the systematic error in the lateral, longitudinal, vertical, and yaw directions in the head-tilted supine position were -0.07 mm, 0.73 mm, -0.63 mm, and -0.01°, respectively, and the standard deviation values of the systematic error were 1.10, 0.97, 0.83, and 0.69, respectively. The mean values of the random error were 0.75 mm, 1.39 mm, 1.02 mm, and 0.57°, respectively, and the standard deviation values of the random error were 0.16, 0.42, 0.28, and 0.26, respectively.

**Table 2.** **Systematic errors (**$\Sigma$**) and Random errors (**$\sigma$**) in the translational (lateral [x-axis], longitudinal [z-axis], and vertical [y-axis]) and rotational (pitch [x-axis], roll [z-axis], and yaw [y-axis]) direction**.

|  | **Supine position** | | | | **Head-tilted supine position** | | | |
| --- | --- | --- | --- | --- | --- | --- | --- | --- |
| **Setup errors** | **Systematic errors (**$\Sigma$**)** | | **Random error(**$\sigma$**)** | | **Systematic errors (**$\Sigma$**)** | | **Random errors (**$\sigma$**)** | |
|  | **Mean (mm)** | **S.D** | **Mean (mm)** | **S.D** | **Mean (mm)** | **S.D** | **Mean (mm)** | **S.D** |
| **Translational** |  |  |  |  |  |  |  |  |
| **Lateral (x-axis) (mm)** | -0.80 | 0.47 | 0.27 | 0.24 | -0.07 | 1.10 | 0.75 | 0.16 |
| **Longitudinal(z-axis) (mm)** | 0.79 | 0.34 | 0.54 | 0.31 | 0.73 | 0.97 | 1.39 | 0.42 |
| **Vertical (y-axis) (mm)** | 0.37 | 0.54 | 0.39 | 0.20 | -0.63 | 0.83 | 1.02 | 0.28 |
| **Rotational** |  |  |  |  |  |  |  |  |
| **Yaw (y-axis) (°)** | 0.17 | 0.56 | 0.35 | 0.17 | -0.01 | 0.69 | 0.57 | 0.26 |

SD=standard deviation

Fig 6 shows the results of the box plot and Mann–Whitney U-test in translation and rotation for the setup errors between the supine and head-tilted supine positions. There was a statistically significant difference in the lateral and vertical directions, and there was no significant difference in the *p*-values of 0.941 and 0.109 in the longitudinal and yaw directions, respectively.

**Fig 6. Boxplot and p-value of the Mann–Whitney U-test in the translation and rotation be-tween the supine and head-tilted supine positions.** Setup errors in the (A) lateral, (B) longitudinal, (C) vertical, and (D) yaw directions.

The results of the box plot and Mann–Whitney U-test for the 3D vector between the supine and head-tilted supine positions are shown in Fig 7. The *p*-value was <0.001, indicating a significant difference.

**Fig 7. Boxplot and *p*-value of the Mann–Whitney U-test in the three-dimensional radiotherapy (3D) vector between the supine and head-tilted supine positions.**

Table 3 shows the CTV to PTV margin calculated by Stroom et al.’s and van Herk et al.’s margin recipe for the systematic errors ($\Sigma$) and random errors ($\sigma$). In the supine position, the CTV to PTV margin was 1.1 mm, 0.9 mm, 1.2 mm, and 1.2° with Stroom et al.’s recipe for lateral, longitudinal, vertical, and yaw directions, and 1.3 mm, 1.1 mm, 1.5 mm, and 1.5° with van Herk et al.’s recipe, respectively. The CTV to PTV margin in the head-tilted supine position was 2.3 mm, 2.2 mm, 1.8 mm, and 1.6° with Stroom et al’s recipe, and 2.9 mm, 2.7 mm, 2.3 mm, and 1.9° with van Herk et al’s recipe, respectively. In the CTV to PTV margin, the head-tilted supine position was larger in all directions than the conventionally supine position.

**Table 3.** **Clinical target volume (CTV) to planning target volume (PTV) margin calculations calculated by Stroom et al.’s [14] and van Herk et al.’s [15] margin recipe from the Systematic errors (**$\Sigma$**) and Random errors (**$\sigma$**).**

| **Series** | **Setup position** | **Image tool** | **Recipe** | **PTV margin** | | | |
| --- | --- | --- | --- | --- | --- | --- | --- |
|  |  |  |  | **Translational** | | **Rotational** | |
|  |  |  |  | **Lateral**  **(mm)** | **Longitudinal**  **(mm)** | **Vertical**  **(mm)** | **Yaw**  **(°)** |
| **Oh et al. [18]** | Supine | CBCT | Van Herk et al | 3.73 | 3.45 | 3.24 |  |
| **Zhou et al [19]** | Supine | MVCT | Stroom et al | 4.8 | 5.0 | 1.5 |  |
| **Oh et al. [12]** | Supine | ExacTrac | Stroom et al | 1.7 | 3.5 | 2.3 | 1.9 |
| **Present Study** | Supine | CBCT | Stroom et al | 1.1 | 0.9 | 1.2 | 1.2 |
|  |  |  | Van Herk et al | 1.3 | 1.1 | 1.5 | 1.5 |
|  | Head tilt supine |  | Stroom et al | 2.3 | 2.2 | 1.8 | 1.6 |
|  |  |  | Van Herk et al | 2.9 | 2.7 | 2.3 | 1.9 |

^＊^Used formula: Stroom et al.’s recipe[14]=$2\Sigma+0.7\sigma$, Van Herk et al.’s recipe [15] = $2.5\Sigma+0.7\sigma$

CBCT=cone-beam computed tomograhy; MVCT= megavoltage computed tomography

# Discussion

In this study, the setup uncertainties and appropriate PTV margins were determined by analysing the setup errors of CBCT images of patients before radiotherapy in the supine position (N=15) and in the head-tilted supine position using a head-tilting baseplate (N=15) in WBRT.

According to the results of our recent study **[6]**, the conformity and homogeneity indices of the target were improved upon using the head-tilting baseplate VMAT in the hippocampal-sparing WBRT, and the mean dose to normal tissues, such as the hippocampus, parotid, right, and left lenses, was significantly reduced. However, one of the limitations of this study was that the dose delivered to the lens was relatively higher in the supine VMAT than in the head-tilted VMAT because the treatment plan was optimised to maximise the target coverage and spare the hippocampus. Therefore, the supine VMAT plan would have been difficult to preserve the hippocampus because the lens constraint was a high priority.

In another study, Miura et al. reported **[7]** that a tilted hippocampus-sparing WBRT with tomotherapy while sparing healthy organs, including the hippocampus and lens, could reduce the treatment time by more than 10%. However, their study had the limitation of a small sample size of five; therefore, a larger cohort study should be conducted. However, because they used two pairs of CT images in the same day, they were able to evaluate two CT images as well as construct a useful deformable image registration algorithm.

Several studies have reported the advantages of the head-tilted supine position in radiotherapy **[6]**,**[7]**,**[8]**,**[9]**; however, no studies have reported on the setup uncertainties and PTV margins for the head-tilted supine position.

The PTV should be extended from the CTV with an appropriate margin. However, less expansion of the CTV could lead to an increase in the uncertainty of the radiation dose to the PTV resulting in undesired radiation treatment outcomes. On the other hand, if the margin of the CTV is expanded too large, a sufficient radiation dose can be delivered to the CTV; however, at the same time, an unintended dose is delivered to the normal tissues **[21]**. Therefore, radiation treatment positions, such as the supine position and head-tilted supine position, which can affect setup uncertainty in radiotherapy, should be investigated for appropriate margin calculations.

Oh et al. **[18]** analysed the setup uncertainties in 21 (438 fractions) cases of brain tumours using daily CBCT. The patient was immobilised in the supine position using a thermoplastic fixation mask, and the registration procedure between the acquired CBCT and the planning CT image was performed according to the bony anatomy. They used van Herk et al. ’s recipe **[15]** to calculate the PTV margin, which was 3.73 mm, 3.45 mm, and 3.24 mm in the lateral, longitudinal, and vertical directions, respectively.

Similar to their study, the PTV margin in the present study using the image tool of CBCT in the supine setup position was 1.3 mm, 1.1 mm, 1.5 mm, and 1.5° with van Herk et al. ’s recipe **[15]**, and 1.1 mm, 0.9 mm, 1.2 mm, and 1.2°, in the lateral, longitudinal, vertical, and yaw directions with Stroom et al. ’s recipe **[14]**, respectively. On the other hand, the PTV margins in the head-tilt setup position were 2.9 mm, 2.7 mm, 2.3 mm and 1.9° with van Herk et al.’s recipe., and 2.3 mm, 2.2 mm, 1.8 mm, and 1.6° with Stroom et al.’s recipe, respectively.

Therefore, when the head-tilted supine position was compared to the conventional supine position in WBRT, it was observed that the PTV should be further expanded by 1.2 mm, 1.3 mm, 0.6 mm, and 0.4° in the lateral, longitudinal, vertical, and yaw directions, respectively as per Stroom et al.’s recipe; moreover, according to van Herk et al.’s recipe, the PTV should be expanded further by 1.6, 1.6, 0.8, and 0.4°, respectively.

Appropriate CTV to PTV margin analysis through systematic and random error analysis can be evaluated using various image tools, such as CBCT **[18]**, MVCT **[19]**, and ExacTrac **[12]**, and the results can be compared as shown in Table 3.

The factors causing differences in the setup uncertainties in the translational and rotational directions according to the setup position in WBRT should be evaluated. As shown in Table 2, it seems that the mask was unable to fix the position of the patient and the baseplate in the head-tilted supine position compared to in the supine position. Therefore, random error is considered larger in the head-tilted supine position. In conclusion, Although the head tilt in the supine position requires a little more margin, it can be applied as a sufficiently safe and effective position in radiotherapy.

This study has certain limitations. First, the patients who received radiotherapy in the supine and head-tilted supine positions did not use the same mask. This is because the treatment position and mask were determined according to our institution’s setup protocol since the setup accuracy varies based on the thermoplastic mask **[22]**,**[23]**. Second, our study analysed images using rigid image registration in the image analysis between the planning CT and pre-treatment verification CBCT; the use of deformable image registration could yields varying results **[24]**,**[25]**,**[26]**,**[27]**. Third, since this study was calculated considering only inter-fractional variation, the PTV margin could be different if intra-fractional variation is included **[28]**,**[29]**,**[30]**. Fourth, since we used only 40° to elevate the patient's head according to our institution's protocol, this study could not demonstrate the effect of different angles on setup uncertainties.

# Conclusions

## This study analysed the setup error of 15 patients treated in the supine position and 15 patients treated in the head-tilted supine position using a head-tilting baseplate in WBRT. Appropriate PTV margins with van Herk et al.’s recipe in WBRT were <2 mm, 1.5° in the supine position, and <3 mm and 2° in the head-tilted supine position in the translational and rotational directions, respectively. Although the head tilt in the supine position requires a little more margin, the head tilt can be applied as a sufficiently safe and effective position in radiotherapy.

# Acknowledgments

This work was supported by the 2021 Yeungnam University Research Grant.

# **References**

1.	McTyre E, Scott J, Chinnaiyan P. Whole brain radiotherapy for brain metastasis. Surgical neurology international. 2013;4(Suppl 4):S236.

2.	Deng X, Zheng Z, Lin B, Su H, Chen H, Fei S, et al. The efficacy and roles of combining temozolomide with whole brain radiotherapy in protection neurocognitive function and improvement quality of life of non-small-cell lung cancer patients with brain metastases. BMC cancer. 2017;17(1):1-9.

3.	Park J, Yea JW. Whole brain radiotherapy using four-field box technique with tilting baseplate for parotid gland sparing. Radiation oncology journal. 2019;37(1):22.

4.	Brown PD, Ahluwalia MS, Khan OH, Asher AL, Wefel JS, Gondi V. Whole-brain radiotherapy for brain metastases: evolution or revolution? Journal of Clinical Oncology. 2018;36(5):483.

5.	Suteu P, Fekete Z, Todor N, Nagy V. Survival and quality of life after whole brain radiotherapy with 3D conformal boost in the treatment of brain metastases. Medicine and pharmacy reports. 2019;92(1):43.

6.	Oh SA, Yea JW, Park JW, Park J. Use of a head-tilting baseplate during volumetric-modulated arc therapy (VMAT) to better protect organs at risk in hippocampal sparing whole brain radiotherapy (HS-WBRT). PloS one. 2020;15(4):e0232430.

7.	Miura K, Kurosaki H, Utsumi N, Sakurai H. Use of a Head-Tilting Baseplate During Tomotherapy to Shorten the Irradiation Time and Protect the Hippocampus and Lens in Hippocampal Sparing-Whole Brain Radiotherapy. Technology in Cancer Research & Treatment. 2021;20:1533033820986824.

8.	Shimizu H, Sasaki K, Aoyama T, Tachibana H, Tanaka H, Koide Y, et al. Examination of the best head tilt angle to reduce the parotid gland dose maintaining a safe level of lens dose in whole‐brain radiotherapy using the four‐field box technique. Journal of Applied Clinical Medical Physics. 2021;22(2):49-57.

9.	Lin Y, Chen P, Shueng P, Lin H, Lai L. Evaluation of various head flexion angles in hippocampal-avoidance whole-brain radiotherapy using volumetric modulated arc therapy. Radiation Physics and Chemistry. 2020;173:108884.

10.	Park J, Park JW, Yea JW. Non–coplanar whole brain radiotherapy is an effective modality for parotid sparing. Yeungnam University journal of medicine. 2019;36(1):36.

11.	Van Herk M, editor Errors and margins in radiotherapy. Seminars in radiation oncology; 2004: Elsevier.

12.	Oh SA, Yea JW, Kang MK, Park JW, Kim SK. Analysis of the setup uncertainty and margin of the daily exactrac 6D image guide system for patients with brain tumors. PloS one. 2016;11(3):e0151709.

13.	Remeijer P, Geerlof E, Ploeger L, Gilhuijs K, van Herk M, Lebesque JV. 3-D portal image analysis in clinical practice: an evaluation of 2-D and 3-D analysis techniques as applied to 30 prostate cancer patients. International Journal of Radiation Oncology* Biology* Physics. 2000;46(5):1281-90.

14.	Stroom JC, De Boer HC, Huizenga H, Visser AG. Inclusion of geometrical uncertainties in radiotherapy treatment planning by means of coverage probability. International Journal of Radiation Oncology* Biology* Physics. 1999;43(4):905-19.

15.	Van Herk M, Remeijer P, Rasch C, Lebesque JV. The probability of correct target dosage: dose-population histograms for deriving treatment margins in radiotherapy. International Journal of Radiation Oncology* Biology* Physics. 2000;47(4):1121-35.

16.	Zhang M, Zhang Q, Gan H, Li S, Zhou S-m. Setup uncertainties in linear accelerator based stereotactic radiosurgery and a derivation of the corresponding setup margin for treatment planning. Physica Medica. 2016;32(2):379-85.

17.	Miao J, Xu Y, Tian Y, Liu Z, Dai J. A study of nonuniform CTV to PTV margin expansion incorporating both rotational and translational uncertainties. Journal of applied clinical medical physics. 2019;20(12):78-86.

18.	Oh YK, Baek JG, Kim OB, Kim JH. Assessment of setup uncertainties for various tumor sites when using daily CBCT for more than 2200 VMAT treatments. Journal of applied clinical medical physics. 2014;15(2):85-99.

19.	Zhou J, Uhl B, Dewit K, Young M, Taylor B, Fei D-Y, et al. Analysis of daily setup variation with tomotherapy megavoltage computed tomography. Medical Dosimetry. 2010;35(1):31-7.

20.	Udayashankar AH, Noorjahan S, Srikantia N, Babu KR, Muzumder S. Immobilization versus no immobilization for pelvic external beam radiotherapy. Reports of Practical Oncology and Radiotherapy. 2018;23(4):233-41.

21.	Oh SA, Yea JW, Kim SK. Statistical determination of the gating windows for respiratory-gated radiotherapy using a visible guiding system. PloS one. 2016;11(5):e0156357.

22.	Gilbeau L, Octave-Prignot M, Loncol T, Renard L, Scalliet P, Grégoire V. Comparison of setup accuracy of three different thermoplastic masks for the treatment of brain and head and neck tumors. Radiotherapy and Oncology. 2001;58(2):155-62.

23.	Sharp L, Lewin F, Johansson H, Payne D, Gerhardsson A, Rutqvist LE. Randomized trial on two types of thermoplastic masks for patient immobilization during radiation therapy for head-and-neck cancer. International Journal of Radiation Oncology* Biology* Physics. 2005;61(1):250-6.

24.	Rigaud B, Simon A, Castelli J, Lafond C, Acosta O, Haigron P, et al. Deformable image registration for radiation therapy: principle, methods, applications and evaluation. Acta Oncologica. 2019;58(9):1225-37.

25.	Paganelli C, Meschini G, Molinelli S, Riboldi M, Baroni G. Patient‐specific validation of deformable image registration in radiation therapy: Overview and caveats. Medical physics. 2018;45(10):e908-e22.

26.	Oh S, Kim S. Deformable image registration in radiation therapy. Radiation oncology journal. 2017;35(2):101.

27.	He Y, Cazoulat G, Wu C, Peterson C, McCulloch M, Anderson B, et al. Geometric and dosimetric accuracy of deformable image registration between average‐intensity images for 4DCT‐based adaptive radiotherapy for non‐small cell lung cancer. Journal of applied clinical medical physics. 2021;22(8):156-67.

28.	Kim SH, Oh SA, Yea JW, Park JW. Prospective assessment of inter-or intra-fractional variation according to body weight or volume change in patients with head and neck cancer undergoing radiotherapy. Plos one. 2019;14(5):e0216655.

29.	Tomihara J, Takatsu J, Sugimoto S, Shikama N, Sasai K. Intrafraction stability using full head mask for brain stereotactic radiotherapy. Journal of Applied Clinical Medical Physics. 2021;22(9):360-70.

30.	Ong CL, Giaj-Levra N, Nicosia L, Figlia V, Tomasini D, Franken EM, et al. Intra-fraction and Inter-fraction analysis of a dedicated immobilization device for intracranial radiation treatment. Radiation Oncology. 2020;15(1):1-7.
