# Supplementary material for: Setup uncertainties and appropriate setup margins in the head-tilted supine position of whole-brain radiotherapy (WBRT)
Source: PLoS One. 2022 Aug 4;17(8):e0271077. doi: 10.1371/journal.pone.0271077 (PMC9352041; doi:10.1371/journal.pone.0271077)
Supplement: Attachment — Submitted filename: Response to Reviewers.docx [file pone.0271077.s002.docx]

| **PONE-D-22-03374**  Setup uncertainties and appropriate setup margins in the head-tilted supine position of whole-brain radiotherapy (WBRT) | |
| --- | --- |
| **Journal Requirements** | **Revisions** |
| 1. Please ensure that your manuscript meets PLOS ONE's style requirements, including those for file naming. | We have revised the manuscript to meet PLOS ONE’s style requirements. |
| 2. We note that you have stated that you will provide repository information for your data at acceptance. Should your manuscript be accepted for publication, we will hold it until you provide the relevant accession numbers or DOIs necessary to access your data. If you wish to make changes to your Data Availability statement, please describe these changes in your cover letter and we will update your Data Availability statement to reflect the information you provide. | We do not have supporting information. Upon submission, we will select and submit appropriately.  The following sentences were revised:  All relevant data are within the manuscript.  (in the Data Availability section) |
| 3. In your Data Availability statement, you have not specified where the minimal data set underlying the results described in your manuscript can be found. PLOS defines a study's minimal data set as the underlying data used to reach the conclusions drawn in the manuscript and any additional data required to replicate the reported study findings in their entirety. | We do not have supporting information. Upon submission, we will select and submit appropriately.  The following sentences were revised:  All relevant data are within the manuscript.  (in the Data Availability section) |
| 4. Please note that funding information should not appear in the Acknowledgments section or other areas of your manuscript. We will only publish funding information present in the Funding Statement section of the online submission form.  Please remove any funding-related text from the manuscript and let us know how you would like to update your Funding Statement. | We removed the funding information from the Acknowledgments section. |
| 5. Please state what role the funders took in the study.  If the funders had no role, please state: "The funders had no role in study design, data collection and analysis, decision to publish, or preparation of the manuscript." | The following sentence was added:  "The funders had no role in the study design, data collection and analysis, decision to publish, or preparation of the manuscript."  (in the Financial Disclosure section) |
| **Reviewer #1** | **Revisions** |
| 1. The objectives of the study were to evaluate the setup uncertainties and determine the appropriate CTV-PTV margins for patients treated with or without a head-tilting baseplate. It is recommended that the abstract and conclusion sections should also include the summaries of setup uncertainty. | Our response: I agree with reviewer’s comments. We added a summary of the results of the setup uncertainty to the abstract and conclusion.  The following sentences were added:  In the c-SP, the means of systematic errors were -0.80, 0.79, and 0.37 mm and random errors were 0.47, 0.34, and 0.54 mm in the lateral, longitudinal, and vertical translational dimensions, respectively. Whereas, for the ht-SP, the means of systematic errors were -0.07, 0.73, and -0.63 mm, and random errors were 0.75, 1.39, 1.02 mm in the lateral, longitudinal, and vertical translational dimensions, respectively.  (in Abstract, red colored sentence)  Also, the following sentences were added:  In the c-SP, the means of systematic errors were -0.80, 0.79, and 0.37 mm and random errors were 0.47, 0.34, and 0.54 mm in the lateral, longitudinal, and vertical translational dimensions, respectively. Whereas, for the ht-SP, the means of systematic errors were -0.07, 0.73, and -0.63 mm, and random errors were 0.75, 1.39, 1.02 mm in the lateral, longitudinal, and vertical translational dimensions, respectively.  (in Conclusion, red colored sentence) |
| 2. The authors have chosen Stroom et al. and van Herk et al. recipes in estimating CTV-PTV margins.  Please mention the rational and limitation (if any) of these selections.  For instance, van Herk et al.’s (2000) method was excluding rotational errors and shape deviations, and considered as a lower limit for safe radiotherapy.  Stroom et al.’s (1999) method was initially applied to prostate, cervix, and lung cancer case. | Our response: We appreciate the reviewer’s appropriate comments. There are many published margin recipes for target, respiration, and organs at risk. We have selected two of the most used margin recipes. The assumptions and relational expressions of this recipe have already been described in the manuscript.  Therefore, we added limitations on the selection of these papers.  The following sentences were added:  Stroom et al. [14] assumed a 95% dose, on an average of 99% of the CTV tested in a practical setting. However, this study has only been demonstrated in prostate, cervix, and lung cancers.  Conversely, Van Herk et al. [15] assumed that the minimum dose for the CTV was 95% for 90% of the patients using the analytical solution for perfect conformation. However, Van Herk et al. reported that because the margin excludes rotational errors and shape deviations, this recipe must be considered as a lower limit for safe radiotherapy.  (in Material and Methods, red colored sentence) |
| 3. Page 14, line 260: The authors have mentioned that the CTV-PTV margins that applied in the clinic were 5 mm. However, the study revealed that the margin should expand further compared to the conventional supine position. Please elaborate further in the discussion section how these findings have changed the current clinical practice if any? | Our response: I agree with reviewer’s comments.  It is necessary to explain how these research results have changed clinical practice. We decided that the head-tilted supine position was safe because it gave a 5 mm margin. In addition, we thought that additional research was necessary.  The following sentences were added:  Therefore, when the ht-SP was compared to the c-SP in WBRT, it was observed that the PTV should be further expanded by 1.2 mm, 1.3 mm, 0.6 mm, and 0.4° in the lateral, longitudinal, vertical, and yaw directions, respectively, as per Stroom et al.’s recipe. Moreover, according to van Herk et al.’s recipe, the PTV should be expanded further by 1.6, 1.6, 0.8, and 0.4°, respectively. In our study, although a little more margin was required in the ht-SP than in the c-SP, our institution extended 5 mm in all directions for expansion of the PTV in the CTV; therefore the ht-SP in WBRT is a sufficiently safe and effective position. Further research is needed to reduce the setup margin in WBRT treatment in the c-SP and ht-SP in the future. |
| 4. Table 1: Please check the table settings (merge/unmerge) of each column titles to avoid confusion. | Our response: we appreciate the reviewer’s appropriate comments. We have changed Table 1 appropriately. |
| 5. Page 6, lines 101: Please check the angle of beam directions. | Our response: We appreciate the reviewer’s appropriate comments. We revised the sentence properly.  The following sentence was added:  3DRT used a beam arrangement in 0°, 90°, 180°, and 270° directions, and the VMAT used two coplanar full-arc beams with clockwise (CW) and counter clockwise (CCW) gantry rotation. |
| 6. Page 8, line 131 and page 9, line 155: The symbols () and () denote different parameter in this manuscript, i.e. random error, systematic error and its standard deviation. Please make necessary amendment in this manuscript to avoid confusion. | Our response: We appreciate the reviewer’s appropriate comments. We have appropriately modified the sentences as follows to avoid confusion.  The following sentences were revised:  where $\boldsymbol{\Sigma}$ is the systematic errors, and $\boldsymbol{\sigma}$ is the random errors. |
| 7. Page 8, line 137: Suggest to move the sentence “All the data were not normally distributed in any direction” to Result section. | Our response: We appreciate the reviewer’s appropriate comments.  The sentence has been moved to the result section. |
| 8. Page 9, line 158 & line 170-181: Please rephrase the paragraphs as some of them were repeatedly mentioned in the text as well as figures 4&5 or Table 2. | Our response: Thank you for the comments. The manuscript contains explanations for Figures 4 and 5 and Table 5. Therefore, it is thought that there may be some repetitive expressions. |
| 9. Table 2’s caption: Please check whether pitch [x-axis] and roll [z-axis] were included in your data set. | Our response: We appreciate the reviewer’s appropriate comments.  The following sentences were revised:  where $\boldsymbol{\Sigma}$ is the systematic errors, and $\boldsymbol{\sigma}$ is the random errors. |
| 10. Table 3: Data for “vertical” should be under “translational” column. | Our response: We appreciate the reviewer’s appropriate comments.  The following portion in Table 3 was revised:   |
| 11. Table 3: Please check the value for “vertical-Stroom recipe- Head tilt supine”, 1.9 instead of 1.8. | Our response: We appreciate the reviewer’s appropriate comments.  The following value was revised (in red font):   |
| 12. Page 15, line 272: Repetition of conclusion “In conclusion, …effective position in radiotherapy.” | Our response: We appreciate the reviewer’s appropriate comments. Repeated sentences have been deleted.  The following sentence was deleted:  ~~In conclusion, Although the head tilt in the supine position requires a little more margin, it can be applied as a sufficiently safe and effective position in radiotherapy.~~ |
| 13. Figure 4-7: the font size of the axis labels was too small. | Our response: We appreciate the reviewer’s appropriate comments. The font size has been increased for Figures 4-7. |
| 14. Reference: Please ensure the reference formatting is in line with the journal requirements, for instance: a. Reference 1: Use the abbreviation “Surg. Neurol. Int.” b. Reference 13: Use the abbreviation “Int. J. Radiat. Oncol. Biol. Phys.” | Our response: We appreciate the reviewer’s appropriate comments. We revised the reference formatting appropriately. |
| **Reviewer #2** | **Revisions** |
| 1. Please explain the differences between with and without a head-tilting base plate. Do you mean the comparison between head-tilting baseplate technique with conventional supine only or is there any other technique involved? | Our response: I agree with reviewer’s comments. The sentence has been revised for clarification.  The following sentences were revised:  Hence, this study evaluated the setup uncertainties and determined the appropriate PTV margins for patients treated in the conventional supine position (c-SP) and head-tilted supine position (ht-SP) using a head-tilting baseplate in WBRT. |
| 2. It would be much better to assigned and acronym these techniques, i.e; c-SP for conventional supine position, hT-SP for head-tilting supine position. | Our response: We agree with reviewer’s comments. Therefore, the entire manuscript has been revised as follows.  The following words were revised:  Conventional supine position (c-SP)  Head-tilted supine position (ht-SP) |
| 3. It would be more relevance to explain the radiotherapy techniques (3DRT and IMRT) applied in the selected patients as opposed to dose fractionation regime.  After all, all patients in this study were treated with 10 fractions treatment. Unless if need to involve any dosimetric comparison, there are no difference in terms of setup uncertainties between 25gy/10# or 30Gy/10#.  The PTV margin and setup uncertainties from these two different techniques would be much appreciated | Our response: We only described the treatment schedule for the patients included in this study in the supine position and the head-tilted supine position. We also indicated the 3D and IMRT techniques ratio.  It is not intended to explain any relevance on this basis.  “Unless if need to involve any dosimetric comparison, there are no difference in terms of setup uncertainties between 25Gy/10# or 30Gy/10#.”  I agree with the reviewer’s comments. |
| 4. Suggest to change ‘3D’ to ‘3DRT’ and ‘IMRT’ to ‘VMAT’ for clearer and more specific description of radiotherapy technique delivery | Our response: I agree with reviewer’s comments. The sentence has been revised for clarification.  The following sentences were revised:  3D→3DRT  IMRT→VMAT |
| 5. It is recommended to briefly explain the CTV delineation, followed by symmetrical/non-symmetrical extension for PTV margin for this study as this is the main objectives of this work.  In addition, author referred to two previous papers, [6] and [10] in which both having different CTV to PTV extension of 5 mm and 7 mm. Please re-direct the reviewer to the correct paper for consistencies. | Our response #1: We appreciate your comment. We added the appropriate sentence for the CTV delineation.  The following sentences were added:  We performed delineation of CT and MRI images of T2-weighted and gadolinium contrast-enhanced T1-weighted sequences using a rigid fused MRI-CT image set. The CTV of the whole brain was defined as the parenchyma and the spinal cord up to the lower level of the atlas.  Our response #2:  The reference [6] mentioned by the reviewer is a 5 mm extension, and the reference [10] mentioned a 7 mm extension.  However, previous papers [6] and [10] are considered to be appropriate references for the treatment plans. |
| 6. Is the 5 mm PTV margin was applied similarly for both 3DRT and VMAT technique? Do state this in your manuscript. | Our response: We appreciate the appropriate comments. We added appropriate sentence for PTV margin.  The following sentences were added:  In the three-dimensional radiotherapy (3DRT) and volumetric-modulated arc therapy (VMAT) techniques, the PTV was created with an extension of 5 mm in all directions from the CTV. |
| 7. Please provide few figures showing the sample of treatment plan with 3DRT beam arrangement (showing the PTV and CTV contouring, with lateral DRR view showing MLC positioning and collimator angle) and VMAT beam arrangement (with DRR showing collimator angle) – for clearer view of both beam arrangement techniques. | Our response: Whole brain radiotherapy using 3DRT and VMAT techniques is a commonly used technique and does not contain any special content in this study. Also, as this study focuses on setup uncertainties rather than patient treatment plans, it is more appropriate to substitute the contents of the manuscript rather than providing figures for beam arrangement and contouring. |
| 8. is it 0, 90, 180 and 270? | Our response: We appreciate the reviewer’s appropriate comments. We revised the sentence to ensure clarity.  The following sentences were added:  3DRT used a beam arrangement in 0°, 90°, 180°, and 270° directions, and the VMAT used two coplanar full-arc beams with clockwise (CW) and counter clockwise (CCW) gantry rotation. |
| 9. Range of collimator angle, photon energy used in the planning, LINAC model used to for treatment, equipped with 6DOF couch? - to be included in the paragraph | Our response: We appreciate the reviewer’s appropriate comments. We revised it to the proper sentence.  The following sentences were added:  The Novalis-Tx (Varian Medical System, CA, USA and BrainLAB, Feldkirchen, Germany) linear accelerator system with a high-definition multi-leaf collimator and 6 degrees of freedom (DOF) robotic couch was used. However, cone-beam CT (CBCT) images in the translational (lateral, longitudinal, and vertical) and rotational (yaw) directions applicable to 4DOF were used. Photon energy of 6 MV was used in all radiation treatment planning. 3DRT used a beam arrangement in 0°, 90°, 180°, and 270° directions, and the VMAT used two coplanar full-arc beams with clockwise (CW) and counter clockwise (CCW) gantry rotation. The collimator of the VMAT was rotated around 290° for CW and 25° for CCW to minimize the tongue-and-groove effect. |
| 10. This sentence and figure is suitable to be in the immobilization & simulation section, rather than delivery techniques section. Suggest to be re-arranged | Our response: I agree with reviewer’s comments. This sentence and figure have been moved to the Immobilisation and CT simulation section. |
| 11. The whole paragraph suggested to be re-write again in manner form.  The section should start with introduction to the institutional setup protocol to include isocentre localization procedure  CBCT imaging parameters used – kV, mAs  Elaborate on pre-treatment CBCT protocol – i.e; what is institutional accepted shift tolerance (< 3mm, 3 degrees?). What is institutional action level, if any (if > 5mm, 5 degrees, did you do re-CBCT and shift or re-setup the patient?)  Elaborate on image registration protocol – window level during matching?using standard clip box? What anatomy should encompass in the clip box? Auto fusion or manual fusion?  Please include the clip box in Figure 2(c) and 3(c)  Any post-shift CBCT images taken? Any post treatment CBCT taken? | Our response: We added appropriate sentences for image registration.  The following sentences were added:  To match the planning CT and CBCT during image registration, an experienced therapist adjusted the window level appropriately. Also, in the image registration, a clip box was used to include PTV. Image registration between the planning CT and CBCT image was performed using bony anatomy auto-matching. There is no institutionally accepted shift tolerance, and all radiation treatment patients were treated with radiation by correcting the translational and rotational directions based on the differences in the planning CT and pre-treatment CBCT.  In addition, the figure does not include a clip box, but a sentence has been added to the manuscript including whether or not the clip box is applied and the scope of clip box.  Unfortunately, studies on post treatment CBCT were not included in this study. We only focused on the setup uncertainties between the planning CT and pre-treatment CBCT images. |
| 12. Figure 2(a) and 3(a)?  Figure 2(b) and 3(b)?  Figure 2(c) and 3(c)? | Our response: We revised it to the proper sentence.  The following sentences were added:  Figures 2a and 3a  Figures 2b and 3b  Figures 2c and 3c |
| 13. Your paragraph should start with explanation on **normality distribution** test first. Then introduced and briefly explain on Van Herks’ and Stroom margin formula calculation prior to other analysis as this is your main study objective. Briefly justified the reason for both formulas incorporated in this study, the significance/advantages of each formula to your study. Lay out all the equation used in this formula.  To calculate the PTV margin either with the use of Van Herks or Stroom’s formula, one should be able to calculate the **individual systematic and random set-up error** for each translational direction. This daily set up error data can be analysed individually for each 30 patients in this study, hence analysis on **inter-fraction analysis** in **all directions** (for both conventional and head-tilted group) can be performed. Please review your raw data to extract the values and perform analysis for this.  Then, briefly explain on the **comparison analysis** between both group (conventional vs head-tilted) calculated using both margin recipe (Van Herk vs Stroom)  Only then, briefly elaborate on 3D vector analysis, the importance/significance of this analysis in your study. | Our response: We revised it to the proper sentence.  The reviewer’s comment is appropriate. According to the reviewer’s opinion, the manuscript has been rearranged to make the clarify.  We introduced and briefly explained van Herks and Stroom margin formula calculations.  In addition, supplementary materials were provided for specific raw data for the individual systematic and random set-up error for each translational direction pointed out by the reviewer.  The following sentences were revised:  Stroom et al. [14] assumed a 95% dose, on an average of 99% of the CTV tested in a practical setting. However, this study has only been demonstrated in prostate, cervix, and lung cancers. Conversely, Van Herk et al. [15] assumed that the minimum dose for the CTV was 95% for 90% of the patients using the analytical solution for perfect conformation. However, Van Herk et al. reported that because the margin excludes rotational errors and shape deviations, this recipe must be considered as a lower limit for safe radiotherapy.  $Stroom et al.^{'}s recipe=2\Sigma+0.7\sigma$  $Van Herk etl al.^{'}s recipe=2.5\Sigma+0.7\sigma$  where $\boldsymbol{\Sigma}$ is the systematic errors, and $\boldsymbol{\sigma}$ is the random errors.  We also compared the 3D vector values between the c-SP and ht-SP. The 3D vector values can be calculated as follows:  $3D vector=\sqrt{x^{2}+y^{2}+z^{2}}$  where x, y, and z are the errors in the lateral, longitudinal, and vertical directions, respectively. |
| 14. Please specify the comparison parameters. Mean individual systematic setup error? Mean individual random setup error? PTV margin? Version? Please state the confidence level. | Our response: it is appropriate comments. We revised it to the proper sentence.  The following sentences were revised:  A non-parametric Mann–Whitney U-test was performed to compare the systematic and random setup errors of the two independent groups between the c-SP and the ht-SP. The mean values of the random and systematic errors are denoted as mean±standard deviation. All the statistical tests were performed using the SPSS statistical software version 22 (SPSS Inc., Chicago, IL, USA), and a *p*-value <0.05 was considered statistically significant. |
| 15. In a manuscript, it’s important for the author to avoid using ‘TRIED TO’. The word showed the lack of confidence of the author in performing your work and justification for the reason to perform this analysis. This gave a negative impression to the reviewer or the reader. | Our response: We revised it to the proper sentence.  The following sentences were revised:  We also compared the 3D vector values between the c-SP and ht-SP. The 3D vector values can be calculated as follows: |
| 16. Justify which of this formula is relevance to your study and sample size. Justify the use of both formulas in your study. Is it for institutional application purpose or act as additional scientific knowledge to the available literatures? | Our response: Your comments is appropriate. The text does not explain that this formula is related to the sample size, this sentence merely explains the assumption of the formula provided by Stroom et a. [14] and van Herk et al [15]. I have edited the sentence for clarity.  The following sentences were revised:  Conversely, Van Herk et al. [15] assumed that the minimum dose for the CTV was 95% for 90% of the patients using the analytical solution for perfect conformation. However, Van Herk et al. reported that because the margin excludes rotational errors and shape deviations, this recipe must be considered as a lower limit for safe radiotherapy. |
| 17. Normalised to what value? Please state in your figure.  Please briefly described your figure data. Insert the X-axis unit. | Our response: In SPSS, we can add normal distribution curves to histograms with ease. Additionally, it quickly shows how (much) the observed distribution deviates from a normal distribution. Therefore, we revised the sentence to clarify.  We inserted the X-axis unit.  The following sentences were revised:  Fig 4. Histograms and normal distribution curves of the setup errors in the translation and rotation for the supine position (c-SP). Setup errors in the (A) lateral, (B) longitudinal, (C) vertical, and (D) yaw directions.  Fig 5. Histograms and normal distribution curves of the setup errors in the translation and rotation for the head-tilted supine position (ht-SP). Setup errors in the (A) lateral, (B) longitudinal, (C) vertical, and (D) yaw directions. |
| 18. In both Van Herk and Stroom formulas, Σ which is the population systematic error defined as the standard deviation (spread) of the individual mean setup errors of the overall population mean, given by;    Hence, only one value will be calculated using this equation without any positive or negative unit. As this is the measure of standard deviation in your data, hence no standard deviation should be calculated. So, how do you get the negative and SD value from this? Or do you mean that you want to present individual set up errors?  For this symbol, 𝜎, again with the same concept as above, do you mean to evaluate the individual random set up error?  I suggest the data to be recalculated again. | As the reviewer said, in both Van Herk and Stroom formulas, Σ is the population systematic error defined as the standard deviation (spread) of the individual mean setup errors of the overall population mean.  In this study, we have studied the appropriate PTV margin from setup uncertainty and applied the most used formula in margin studies.  Therefore, it is considered appropriate to apply this formula to this study. |
| 19. No data pertaining to these axes. As I recalled in all paragraph above, no analysis done for these axes. Please delete. | Our response: Thank you for the comment.  The following sentences were revised:  **Table 2.** **Systematic errors (**$\Sigma$**) and random errors (**$\sigma$**) in the translational (lateral [x-axis], longitudinal [z-axis], and vertical [y-axis]) and rotational (yaw [y-axis]) directions** |
| 20.Only one Y-axis should be scale. Delete the scale on the right side of Y axis.  What kind of setup errors? Is it the individual systematic setup error or the individual random setup error? Please be specific. | Our response: I believe your comment is justified.  Accordingly, the scale on the right side of the Y-axis has been deleted.  Figure 6 shows the setup error between the supine and head-tilted supine positions as a box plot. It seems that it cannot be concluded that it is a systematic error or a random error specifically mentioned by the reviewer. Therefore, we believe that it is appropriate to express our thoughts as a setup error. |
| 21. Only one Y-axis should be scale. Delete the scale on the right side of Y axis | Our response: Per your comment, the scale on the right side of the Y-axis has been deleted. |
| 22. Since, there is uncertainties in the above calculated population systematic and random errors data in this study, I suggest the finalized PTV margin to be calculated again for each direction.  Recalculation of the PTV margin based on the data provided in Table 2 in this manuscript, however didn’t resulted in the same PTV margin values as shown in Table 3 of this manuscript. Reconfirmation of the correct formula used and correct calculation is needed. | Our response: We appreciate the reviewer’s appropriate comments.  Thank you for your concerns and comments.  We reviewed and confirmed the raw data studied. Also, the formula was recalculated. It has been corrected because one non-rounded value was included below.  The following value was revised (in red font):   |
| 23. Change to Studies  Change to Imaging modalities  Vertical should be under ‘Translational’ column. Please re-arrange it  tomography | Our response: We appreciate the reviewer’s appropriate comments. Following the reviewer’s recommendation, the following sections (in red font) were revised.   |
| 24. I don’t see the relevance of discussing the advantages of head-tilted in terms of dose sparing in this manuscript, as these has been discussed in the authors’ previous paper.  In addition to this, in this current manuscript, as shown in Table 1, the head-tilted WBRT was treated using 3DRT technique instead of VMAT technique, which contradict to whatever has been discussed in this paragraph.  It is advisable to discuss on the impact of different imaging modalities used as image guidance treatment. | Talking about the advantages of head-tilt in terms of dose sparing in this manuscript is the motivation for this study. Therefore, I think it is appropriate to briefly explain the reason for performing head-tilted WBRT in the discussion.  In this study, it is not the goal of to look at the difference between VMAT and 3DRT technology. The goal of this study is to analyze the setup error according to the patient’s setup position and obtain the appropriate PTV margin accordingly.  Furthermore, research on the effects of various imaging techniques used as image-guided radiotherapy will be further analyzed. |
| 25. Is there any comparison analysis for this? Is the difference between this position is significant? Suggest to perform the comparison analysis | This study analyzed setup errors of CBCT images of patients before radiotherapy in the c-SP (N=15) and ht-SP using a head-tilting baseplate (N=15) in WBRT.  Furthermore, this study was performed to determine whether there is a difference between head-tilt and conventional supine position. |
| 26. To the best of my knowledge, the PTV margin calculated only involved the translational coordinate, which is practical to be implemented when expanding the CTV to PTV in the treatment planning system. It is unlikely to expand the PTV margin for rotational coordinate  The analysis of rotational usually were made to evaluate the dosimetric impact of PTV rotation. You can find literatures pertaining to this which can be added as part of your study analysis and discussion | As the reviewer said, in the radiation treatment planning system, the extension of the CTV to PTV margin is extended in the translational coordinate direction.  However, during online setup matching, it is possible to move in the translation (x, y, z direction) and rotation (yaw direction).  In addition, the difference in the rotational as well as the translation direction in the conventional supine and the head-tilted supine position is one of the points of interest.  Therefore, it is meaningful to analyze the setup error not only in the translation but also in the rotation direction. |
| 27. Prior to the study limitation, please discuss on;  1) The reason for differences in PTV margin calculated in this study with other studies as shown in Table 3 – this should be discussed for validity of the data in this study  The results of 3D vector calculated in this study – any comparison to other study? The significance of 3D vector calculation? | 1) The PTV margin calculated in this study compared with the results of other previous studies is shown in Table 3.  As shown in the manuscript, these PTV margins appear differently for each institution due to various systematic and random errors. These results indicated that the existing manuscripts were also compared.  The following sentence were revised:  Oh et al. **[18]** analysed the setup uncertainties in 21 (438 fractions) cases of brain tumours using daily CBCT. The patient was immobilised in the c-SP using a thermoplastic fixation mask, and the registration procedure between the acquired CBCT and the planning CT image was performed according to the bony anatomy. They used van Herk et al.’s recipe **[15]** to calculate the PTV margin, which was 3.73 mm, 3.45 mm, and 3.24 mm in the lateral, longitudinal, and vertical directions, respectively.  Similar to their study, the PTV margin in the present study using the image tool of CBCT in the c-SP was 1.3 mm, 1.1 mm, 1.5 mm, and 1.5° with van Herk et al.’s recipe **[15]**, and 1.1 mm, 0.9 mm, 1.2 mm, and 1.2°, in the lateral, longitudinal, vertical, and yaw directions with Stroom et al.’s recipe **[14]**, respectively. Whereas the PTV margins in the ht-SP were 2.9 mm, 2.7 mm, 2.3 mm and 1.9° with van Herk et al.’s recipe., and 2.3 mm, 2.2 mm, 1.9 mm, and 1.6° with Stroom et al.’s recipe, respectively.  2) In this study, 3D Vector was defined and calculated as follows.  $3D vector=\sqrt{x^{2}+y^{2}+z^{2}}$  In this study, 3D vector values with other studies were not compared, but the setup error in the conventional supine setup position and head-tilted supine position, which is the goal of this study, was compared using 3D Vector. |
| 28. Do you conclude that the institutional 5 mm margin is enough? or too big? Any change in institutional applied margin from this study? Please present a concrete conclusion derived from this study. | As the conclusion of this study has already been described, the setup error was analyzed in the supine and head-tilting supine position. Calculated using van Herk et al's recipe, which is the most used in margin recipe, <2 mm and 1.5° in the supine position and <3 mm and 2° in the head-tilting supine position, the head tilting supine position is a sufficiently safe and effective position in radiotherapy. |
| **Reviewer #3** | **Revisions** |
| 1. The write-up has too many short paragraphs of single/two sentences that can be combined with other paragraphs. | The entire manuscript was checked and revised. |
| 2. Abstract: The motivation of using head-tilting technique can be briefly highlighted in the abstract | Our response: We appreciate the reviewer’s appropriate comments.  The following sentences are added:  Various applications of head-tilting techniques in whole-brain radiotherapy (WBRT) have been introduced. However, a study on the setup uncertainties and margins in head-tilting techniques has not been reported. This |
| 3.Line 23: Please rephrase the sentence. The sentence is describing the image correction not the radiotherapy delivery. “Radiotherapy was performed by correcting the translational (lateral, longitudinal, and vertical) and rotational (yaw) errors based on the differences in computed tomography (CT) and cone-beam CT.” | Our response: We appreciate the reviewer’s appropriate comments.  The following sentences are revised:  Daily setup verification using planning computed tomography (CT) and cone-beam CT was corrected for translational (lateral, longitudinal, and vertical) and rotational (yaw) errors. |
| 4. Suggest to compare results obtained with recently published margin, e.g., <http://doi.org/10.1002/acm2.13291> | Our response: Abubakar et al. have developed a practical and automated algorithm for the calculation of setup error correction.  The algorithm also allows for automated calculation of the population setup errors and PTV margin for the no correction protocol, NAL, and eNAL offline protocols from the same dataset.  The algorithm would select the most effective protocols in terms of setup error and PTV margin reduction for a specific anatomic site for a radiotherapy center.  However, for example, the recommended paper was different from this study for head and neck patients; therefore, it is difficult to compare it with this study.  Furthermore, our findings have already been specifically compared in Table 3 comprehensively with references [12], [18], [19]. |
| 5. Line 272, In discussion, although the head tilt gives larger margin, perhaps authors can comment on how the benefit of sparing OAR, can justify the use of head-tilt? This can be included in the conclusion briefly. | Our response: we appreciate the reviewer’s appropriate comments.  The following sentences are added:  Although the head-tilt in the supine position requires a little more margin, it can be utilized as a safe and effective position in radiotherapy. Therefore, additional studies are needed on the advantages of sparing organs at risk and the disadvantages of a little more margin in the head-tilt supine position. |
| 6.Perhaps authors can discuss how the head-tilt technique can be improved, to reduce the uncertainties. | Our response: We appreciate the reviewer’s appropriate comments. The result of this study was that a little more margin was required in the head-tilt supine position than in the conventional supine position. Therefore, further studies are needed to reduce uncertainties in the head-tilt supine position.  The following sentences are added:  In our study, although a little more margin was required in the ht-SP than in the c-SP, our institution extended 5 mm in all directions for expansion of the PTV in the CTV; therefore, the ht-SP in WBRT is a sufficiently safe and effective position. Further research is needed to reduce the setup margin in WBRT treatment in the c-SP and ht-SP in the future. |
